# Microsatellite cross-species amplification and utility in southern African elasmobranchs: A valuable resource for fisheries management and conservation

**DOI:** 10.1186/1756-0500-7-352

**Published:** 2014-06-10

**Authors:** Simo N Maduna, Charné Rossouw, Rouvay Roodt-Wilding, Aletta E Bester-van der Merwe

**Affiliations:** 1 Department of Genetics, Stellenbosch University, Private Bag X1, Matieland 7602, South Africa

**Keywords:** Cross-species amplification, Microsatellites, Multiplex assays, Genetic diversity, Species identification, Conservation management

## Abstract

**Background:**

Similarly to the rest of the world, southern Africa’s diverse chondrichthyan fauna is currently experiencing high fishing pressures from direct and non-direct fisheries to satisfy market demands for shark products such as fins and meat. In this study, the development of microsatellite markers through cross-species amplification of primer sets previously developed for closely related species is reported as an alternative approach to *de novo* marker development. This included the design of four microsatellite multiplex assays and their cross-species utility in genetic diversity analysis of southern African elasmobranchs. As this study forms part of a larger project on the development of genetic resources for commercially important and endemic southern African species, *Mustelus mustelus* was used as a candidate species for testing these multiplex assays in down-stream applications.

**Results:**

Thirty five microsatellite primer sets previously developed for five elasmobranch species were selected from literature for testing cross-species amplification in 16 elasmobranch species occurring in southern Africa. Cross-species amplification success rates ranged from 28.6%-71.4%. From the successfully amplified microsatellites, 22 loci were selected and evaluated for levels of polymorphism, and four multiplex assays comprising of the 22 microsatellites were successfully constructed, optimised and characterised in a panel of 87 *Mustelus mustelus* individuals. A total of 125 alleles were observed across all loci, with the number of alleles ranging from 3–12 alleles. Cross-species amplification of the four optimised multiplex assays was further tested on 11 commercially important and endemic southern African elasmobranch species. Percentage of polymorphism ranged from 31.8%-95.5% in these species with polymorphic information content decreasing exponentially with evolutionary distance from the source species.

**Conclusions:**

Cross-species amplification of the 35 microsatellites proved to be a time- and cost-effective approach to marker development in elasmobranchs and enabled the construction of four novel multiplex assays for characterising genetic diversity in a number of southern African elasmobranch species. This study successfully demonstrated the usefulness of these markers in down-stream applications such as genetic diversity assessment and species identification which could potentially aid in a more integrative, multidisciplinary approach to management and conservation of commercially important cosmopolitan and endemic elasmobranch species occurring in southern Africa.

## Background

The subclass Elasmobranchii (sharks, skates and rays) comprises a diverse group of over 1000 species, and is representative of one of the most ancient extant vertebrate lineages [1]. Recently, pressures from direct and non-direct fisheries have resulted in the depletion of elasmobranch populations globally [2]. Decline in wild populations of elasmobranchs is further compounded by their life history traits that are more similar to those of mammals (*e.g*. low fecundity, late maturity and long gestation periods) than those of teleost fishes [3,4]. In comparison, elasmobranchs may not respond well to the high fishing pressures. This trend has been particularly pronounced for sharks due to unregulated harvesting to support an increase in demand for shark products (*e.g*. fins, meat, liver oil, skin and cartilage). A drastic reduction in population size (population bottleneck) can result in small populations experiencing the accumulating effects of inbreeding leading to severe loss of genetic diversity [5,6]. These trends have previously been reported for species such as the basking shark (*Cetorhinus maximus*) [7] and the narrownose smoothhound shark (*Mustelus schmitti*) [8]. Assessing genetic diversity and population structure of wild populations is therefore important for sustainable long-term management of the global shark fishery industry.

Misidentification of shark species in fisheries operations is also a widespread concern [9-12], and molecular individual identification methods have been developed to alleviate this problem [4,13-17]. To integrate genetic knowledge with fisheries management, it is imperative for shark fisheries to report shark landings by species instead of lumping them into species- or family groups (*e.g.* houndsharks, carcharinids, hammerheads *etc*.). This stems from the difficulties involved with unambiguously identifying species within and across families [13,18]*e.g*., carcharinids (*Carcharhinus brachyurus*, *C. obscurus* and *C. plumbeus*) and houndsharks (*Mustelus mustelus*, *M. palumbes* and *Galeorhinus galeus*) due to a high degree of conserved interspecific morphology [14]. Neglecting to report shark landings by species overlooks important differences in species susceptibility and population vulnerability to exploitation [15], and that in turn has important implications for species-specific conservation, management and trade monitoring programmes [19].

The general lack of molecular genetic markers (*e.g*. microsatellites) for many elasmobranch species impedes population and conservation genetic studies in that these markers can provide valuable information relating to population dynamics (spatial and temporal genetic variation) of individual species. Microsatellites are highly polymorphic due to their high mutation rate (between 10^-3^ and 10^-4^ mutations per gamete per generation) resulting in extensive length polymorphism [20,21]. This makes microsatellite markers one of the most powerful molecular genetic tools with a remarkable array of applications ranging from genetic diversity [22,23] and population structure inference [24,25] to discerning genetic mating systems [26,27] and the identification of species [28-30].

Because the *de novo* development of microsatellites is challenging due to notoriously low rates of polymorphism in elasmobranchs [31], the development of microsatellite markers through cross-species amplification is the most effective alternative approach to *de novo* development of microsatellites and has recently also been reported in sharks [32]. Microsatellite cross-species amplification relies on the presence of conserved microsatellite flanking sequences [33], which in some organisms markedly demonstrate a high degree of conservation following millions of years of divergent evolution (*e.g*., 250 million years in sharks [28] and 470 million years in fish [34]). The success rate of microsatellite cross-species amplification has directly been correlated to the evolutionary distance between the source species and the target species [33,35].

The underrepresentation of endemic taxa in many cross-species amplification studies is unfortunate as endemics should be of great interest for conservation of biodiversity on a regional scale. Southern Africa has one of the most diverse chondrichthyan faunas in the world, consisting of some 181 species in 44 families of which 34 species are endemic to southern Africa [36,37]. Growing concerns regarding the sustainability of the southern African shark fishery, stemming from the local declines of cosmopolitan and endemic species, lead to stricter regulations being imposed so as to avert the collapse of natural populations [38-40]. Accordingly, we report here the development of microsatellite markers through cross-species amplification of species-specific primers from closely related species. This included the design and optimisation of four microsatellite multiplex assays and their cross-species utility in genetic diversity analysis of 11 southern African elasmobranch species.

## Results and discussion

### Cross-species amplification

**Figure 1:**
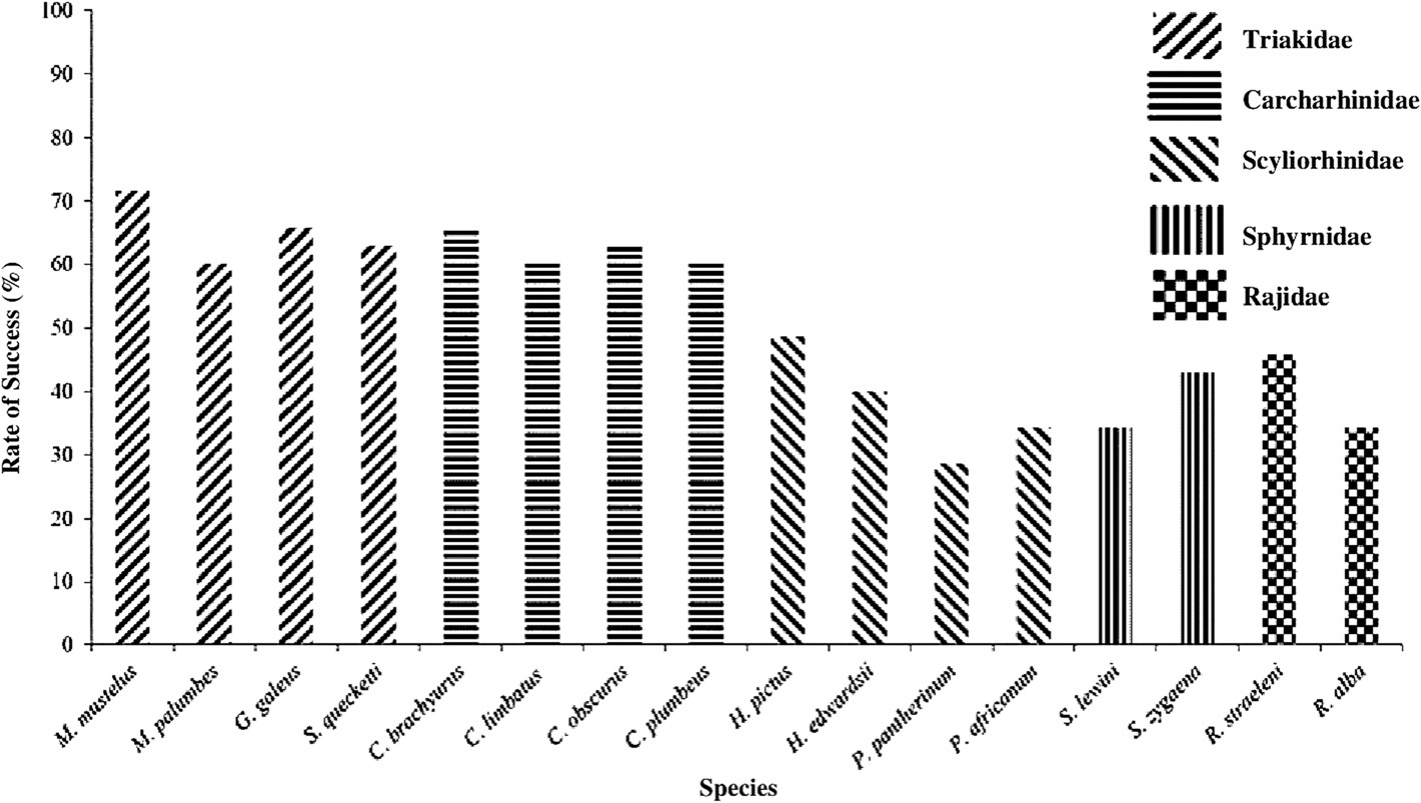
Success rates of 35 microsatellite loci across five families of southern African elasmobranch species.

**Figure 2:**
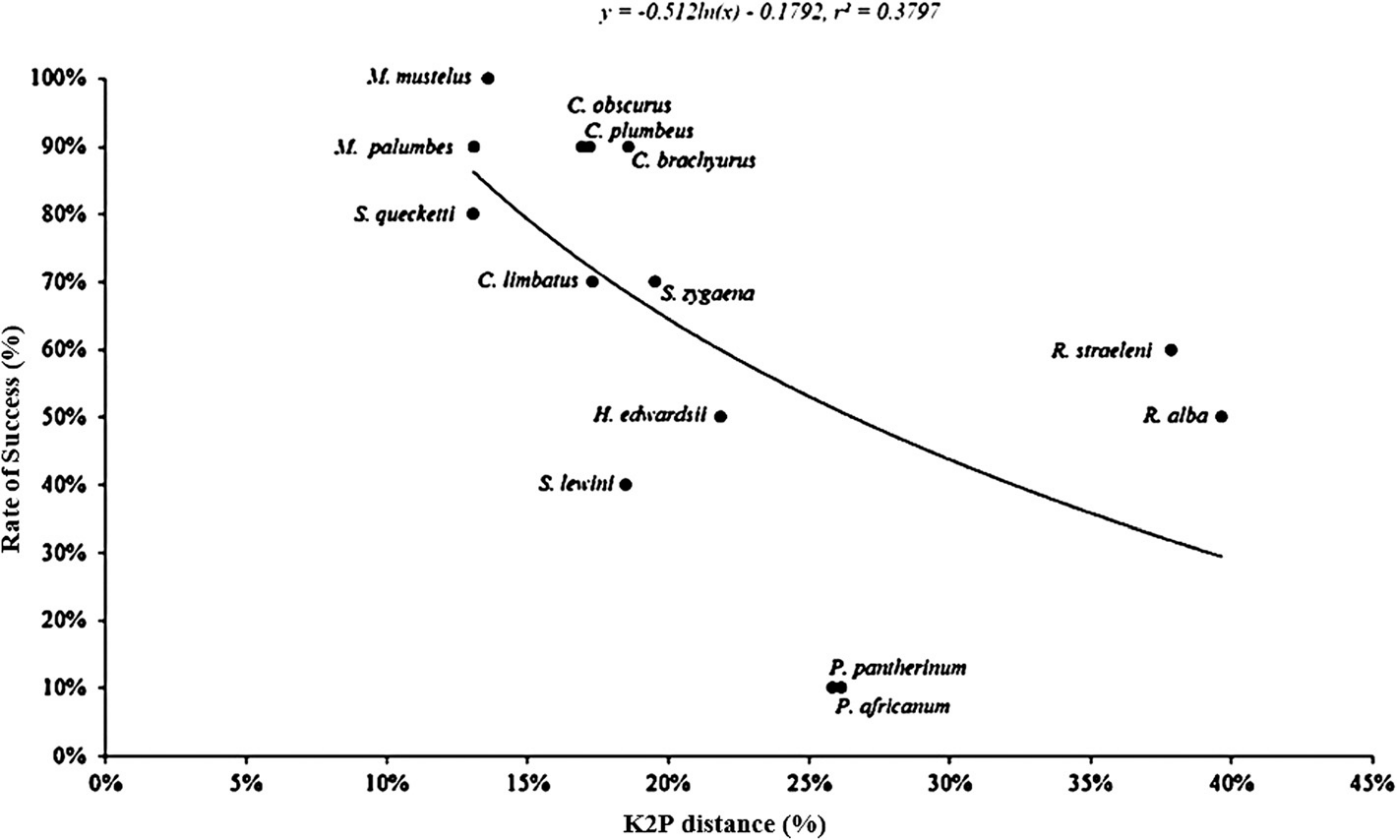
**Cross-species amplification performance of *Galeorhinus galeus *** **microsatellites in 15 of the 16 elasmobranch species, and genetic divergence between *G. galeus *** **and the target species based on *ND2 *** **sequences.**

**Figure 3:**
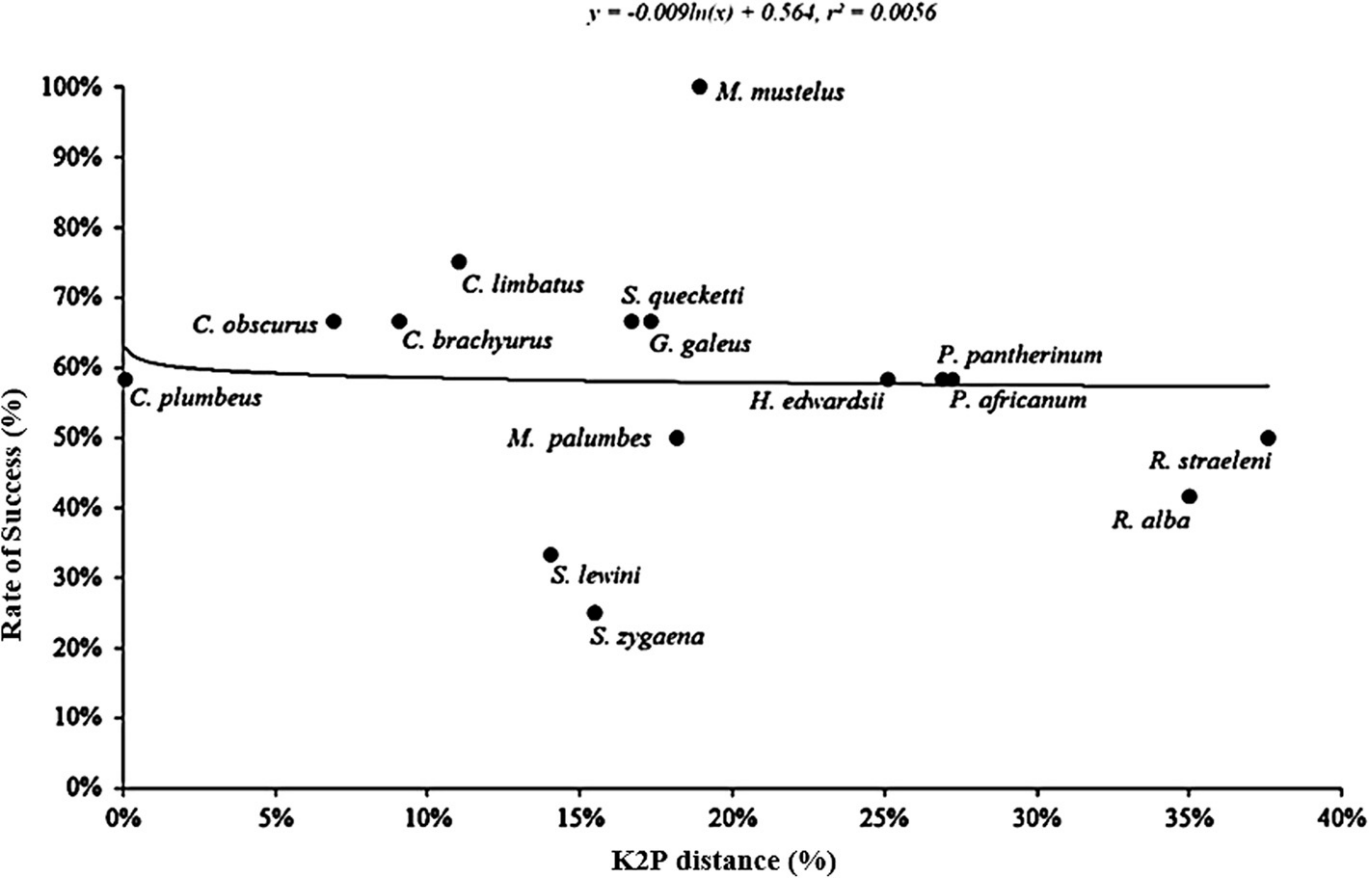
**Cross-species amplification performance of *Mustelus canis *** **microsatellites in 15 of the 16 elasmobranch species, and genetic divergence between *M. canis *** **and the target species based on *ND2 *** **sequences.**

Development of microsatellite loci through cross-species amplification proved useful in establishing genetic markers for shark species that are commercially important and those (typically endemics) that are indirectly affected by fisheries’ operations. Amplification of the 35 microsatellites in 50 individuals from 16 different elasmobranch species (1–4 individuals per species) proved to be effective (Table 1). Cross-species amplification success rates or the percentage of microsatellites that amplified successfully ranged from 60.00%-71.40% in the Triakidae and Carcharhinidae families and from 28.57%-48.57% in the Scyliorhinidae, Sphyrnidae and Rajidae families (Figure 1). The higher success rates in the Triakidae and Carcharhinidae is expected as most microsatellites tested in this study were originally developed for species within the Triakidae family. Overall, the microsatellites showed less successful cross-species amplification to the taxa more divergent from the source species. Notably none of the individuals showed PCR amplification at any of the six *Scyliorhinus canacula* microsatellites. This may in part be attributed to *S. canacula* being more distantly related to the study species. The mean genetic distance between the taxa was 21.4 ± 1.7% (mean ± SD) (*G. galeus* as source species; Figure 2) and 18.7 ± 1.5% (*M. canis* as source species; Figure 3). *Haploblepharus pictus* could not be represented in the distance plot due to the lack of genetic information available in GenBank and Global Cestode Database: Elasmobranchs Specimens.

**Table 1:** Cross-species amplification of the 35 microsatellites among 16 elasmobranch species of southern Africa

**Species**	**References**	**MM**	**MP**	**GG**	**SQ**	**CB**	**CL**	**CO**	**CP**	**HP**	**HE**	**PP**	**PA**	**SL**	**SZ**	**RS**	**RA**
**Loci**		**(*n*** **= 4)**	**(n = 3)**	**(*n*** **= 4)**	**(*n*** **= 1)**	**(*n*** **= 4)**	**(n = 3)**	**(*n*** **= 4)**	**(*n*** **= 4)**	**(*n*** **= 4)**	**(*n*** **= 4)**	**(n = 4)**	**(*n*** **= 1)**	**(n = 3)**	**(n = 3)**	**(*n*** **= 1)**	**(*n*** **= 3)**
*Mh1*	[56]	+	+	+	+	+	+	+	+	+	+	+	+	+	+	+	++
*Mh2*	[57]	+	+	+	+	+	+	+	+	-	-	-	-	+	+	-	-
*Mh6*	[56]	-	-	+	-	+	-	-	-	-	-	-	-	-	-	-	-
*Mh9*	[57]	+	+	+	+	+	-	+	+	-	-	-	-	-	-	-	-
*Mh25*	[56]	+	+	+	+	+	+	+	+	+	+	+	+	-	+	+	++
*Mca25*	[49]	+	-	-	-	+	+	++	++	+	-	+	+	-	-	+	+
*Mca31*	[49]	-	-	-	+	+	+	+	-	-	-	-	-	-	+	-	-
*Mca33*	[49]	+	+	+	+	+	+	+	-	+	+	+	+	-	-	+	+
*Mca44*	[49]	++	-	+	-	-	-	-	-	+	+	+	+	-	-	+	++
*McaB5*	[49]	+	+	+	+	+	+	+	+	+	+	+	+	+	-	+	+
*McaB6*	[49]	+	-	+	+	+	+	+	+	+	+	-	-	+	-	-	-
*McaB22*	[49]	+	+	+	+	-	+	+	+	+	+	+	+	-	+	+	-
*McaB27*	[49]	+	+	+	+	+	-	-	+	-	+	-	-	-	+	-	-
*McaB33*	[49]	-	-	-	-	+	+	+	+	-	-	-	-	+	-	-	-
*McaB35*	[49]	+	+	-	+	-	-	-	-	+	-	+	+	-	-	-	++
*McaB37*	[49]	+	+	+	+	-	+	-	-	-	-	-	-	+	-	-	-
*McaB39*	[49]	+	-	+	-	+	+	+	+	+	+	+	+	-	-	+	-
*Gg2*	[50]	+	+	+	+	+	-	-	-	+	-	-	-	-	+	++	+
*Gg3*	[50]	+	+	+	+	+	+	+	+	+	+	-	-	+	-	++	+
*Gg7*	[50]	+	+	+	-	+	-	+	+	-	+	-	-	-	+	-	+
*Gg11*	[50]	+	+	+	++	++	+	++	++	+	+	-	-	-	+	-	-
*Gg12*	[50]	+	+	+	-	+	-	+	+	+	-	-	-	-	-	-	-
*Gg15*	[50]	+	+	+	+	+	+	++	++	+	+	+	+	+	+	+	-
*Gg17*	[50]	+	-	+	+	+	+	+	+	-	+	-	-	+	-	-	+
*Gg18*	[50]	+	+	+	+	+	+	++	+	+	-	-	-	-	++	+	+
*Gg22*	[50]	+	+	+	+	+	+	+	+	+	-	-	-	-	++	+	-
*Gg23*	[50]	+	+	+	+	+	+	+	+	-	-	-	-	+	++	+	+
*Rp16-nfrdi*	[44]	+	++	-	+	+	++	+	+	-	-	-	+	++	+	+	++
*Rp35-nfrdi*	[44]	-	+	-	++	-	+	-	-	-	-	-	+	+	++	+	++
*Scan02*	[45]	-	-	-	-	-	-	-	-	-	-	-	-	-	-	-	-
*Scan06*	[45]	-	-	-	-	-	-	-	-	-	-	-	-	-	-	-	-
*Scan12*	[45]	-	-	-	-	-	-	-	-	-	-	-	-	-	-	-	-
*Scan14*	[45]	-	-	-	-	-	-	-	-	-	-	-	-	-	-	-	-
*Scan15*	[45]	-	-	-	-	-	-	-	-	-	-	-	-	-	-	-	-
*Scan16*	[45]	-	-	-	-	-	-	-	-	-	-	-	-	-	-	-	-

–, no visible band or faint bands with insufficient band intensity for scoring alleles were observed; +, solid bands with sufficient intensity for scoring alleles were detected; ++, solid bands with artefacts were produced but with at least one band of expected allele size. *Mustelus mustelus* (MM), *Mustelus palumbes* (MP), *Galeorhinus galeus* (GG), *Scylliogaleus quecketti* (SQ), *Carcharhinus brachyurus* (CB), *Carcharhinus limbatus* (CL), *Carcharhinus obscurus* (CO), *Carcharhinus plumbeus* (CP), *Haploblepharus pictus* (HP), *Haploblepharus edwardsii* (HE), *Poroderma africanum* (PA), *Poroderma pantherinum* (PP) *Sphyrna lewini* (SL), *Sphyrna zygaena* (SZ), *Raja straeleni* (RS) and *Raja alba* (RA).

Results of cross-species amplification performance of *G. galeus* microsatellites exhibited a logarithmic regression function (Figure 2), albeit non-significant (slope within the 95% CI for no difference from zero), that may explain the general trend of negative correlation between cross-species amplification performance and genetic divergence seen across taxa [30,33,35,41]. Cross-species amplification of microsatellite markers from source to target species is generally negatively correlated with evolutionary divergence [30,42-45]. A similar trend was not observed for the *M. canis* microsatellites (Figure 3). This can be due to different life history traits (*i.e*. mating system and generation time) and genome size (*C* value) between the source and target species, which have been previously found to have significant negative effects on cross-species amplification success [33]. However, apart from the source-target species evolutionary distance other factors, such as mutations in microsatellite flanking sequences, may affect the success rate of cross-species amplification. Since microsatellites are usually found in non-coding regions where the substitution rate is higher than in coding regions [46], these microsatellite flanking sequences which serve as regions for PCR primer design and binding sites are prone to mutations [35]. Mutations (indels) in these regions may therefore result in null alleles and in turn affect the patterns of cross-species amplification as demonstrated in birds [47] and salmonids [48].

Additionally, *M. canis* microsatellite loci were isolated from an enriched genomic library [49] whereas for *G. galeus* the microsatellites were developed by a high-throughput sequencing approach (Roche 454 pyrosequencing) [50]. Based on the observed data it is hypothesised that these different approaches may have influenced the cross-species performance possibly due to the different resolving power of each approach for capturing microsatellites distributed across different parts of the genome. Castoe *et al*. [51] argues that enrichment-based approaches commonly use a few specific repeated motifs, which are largely selected without prior knowledge of their abundance in the genome and therefore could introduce potential bias in genome representativeness. In contrast, microsatellite identification from randomly sequenced genomic regions (*e.g*. Roche 454 NGS) allows for an unbiased assessment of all types of microsatellite loci present in a genome [51].

Cross-species amplification of orthologous microsatellites, due to the presence of conserved microsatellite flanking sequences [33], can persist over millions of years following divergent evolution as previously shown in sharks (250 million years [28]) and in fish (470 million years [34]). This indicates lower mutation rates within microsatellite flanking regions in aquatic organisms [30,34]. The reported cross-amplified microsatellite markers will thus contribute to establishing a molecular genetic marker repository for each of the Southern Africa elasmobranchs species included in this study. Future research efforts may be dedicated to generating microsatellite primers that have a high cross-species utility (*e.g*. [52]) as well as *in silico* mining of polymorphic microsatellite markers from expressed sequence tag data [53].

### Multiplex assay characterisation

Twenty-two microsatellite loci that successfully cross-amplified across the study species and showed polymorphism in initial screening tests were used to develop four multiplex assays (MPS) comprised of at least five microsatellites each. These multiplex assays were characterised in a panel of 87 common smoothhound sharks (*Mustelus mustelus*) (Table 2). All 22 microsatellite loci were polymorphic across the multiplexes. With the exception of one locus, *McaB22*, all the microsatellite loci were found to deviate significantly from Hardy-Weinberg equilibrium most likely due to Wahlund effect as samples were pooled from diverse geographical locations for analysis. MicroChecker detected no significant genotyping errors but indicated that null alleles were present at two loci (*Mh9* and *Gg7*). Slatkin’s exact test for neutrality indicated that two loci (*McaB22* and *Gg3*) were candidates for being subjected to selection.

**Table 2:** **Characterisation of four multiplex assays for *Mustelus mustelus *** **based on 87 individuals from southern Africa**

**Locus**	**Microsatellite repeat motif**	**[P]**	**Dye**	**Size range (bp)**	***A***_***N***_	***A***_***E***_	***H***_***O***_	***H***_***E***_	***PIC***	***F***_***IS***_	***Fr***_***NULL***_	***P***_***E-W***_
*Mh1*	(AG)_n_	0.2	VIC	191-211	7	2.2	0.885	0.544	0.443	-0.633**	-0.223	0.931
*Mh2*	(GA)_9_	0.3	VIC	587-597	4	1.7	0.367	0.402	0.342	0.089**	0.023	0.688
*Mh9*	(GA)_9_	0.4	FAM	312-326	5	1.7	0.337	0.429	0.373	0.214**	0.062 ^*b*^	0.723
*Mh25*	(CT)_n_	0.2	FAM	122-148	8	1.6	0.356	0.404	0.385	0.118**	0.032	0.802
*Mca25*	(CA)_n_(CT)_n_	0.2	PET	232-240	3	1.9	0.563	0.463	0.382	-0.217**	-0.070	0.226
*McaB39*	(CA)_10_GAT(AC)_8_	0.2	NED	177-212	3	2.0	1.000	0.509	0.384	-0.977**	-0.328	0.501
**MPS1 (mean)**	**-**	**-**	**-**	**-**	**5**	**1.9**	**0.585**	**0.459**	**0.385**	**-0.234**	**-0.084**	**0.645**
*McaB5*	(GT)_11_	0.2	VIC	189-210	10	3.5	0.826	0.716	0.674	-0.155*	-0.067	0.330
*McaB6*	(CA)_10_	0.2	FAM	226-266	9	3.3	0.756	0.702	0.655	-0.077*	-0.034	0.498
*McaB22*	(AC)_18_	0.2	NED	137-179	12	8.2	0.874	0.882	0.865	0.010	0.002	0.002
*McaB27*	(GT)_6_	0.2	PET	138-199	4	2.1	0.965	0.536	0.424	-0.808**	-0.282	0.589
*Mca33*	(ATC)_5_	0.2	FAM	189-199	6	3.0	0.872	0.674	0.609	-0.295**	-0.121	0.347
*McaB37*	(GT)_5_	0.2	NED	219-251	11	1.9	0.483	0.486	0.431	0.007**	-0.016	0.997
**MPS2 (mean)**	**-**	**-**	**-**	**-**	**9**	**3.7**	**0.796**	**0.666**	**0.610**	**-0.220**	**-0.086**	**0.461**
*Gg2*	(TG)_n_	0.2	NED	249-259	7	3.2	1.000	0.688	0.632	-0.458**	-0.188	0.324
*Gg3*	(GATT)_n_	0.2	PET	257-265	2	2.0	1.000	0.503	0.375	-1.000**	-0.333	0.001
*Gg7*	(AG)_n_	0.2	NED	296-312	4	1.6	0.310	0.393	0.343	0.212**	0.058^*b*^	0.584
*Gg11*	(TCCC)_n_	0.2	NED	329-363	4	1.2	0.061	0.182	0.173	0.666**	0.000	0.792
*Gg12*	(TA)_n_	0.2	FAM	276-296	4	1.8	0.610	0.454	0.361	-0.347**	-0.110	0.807
**MPS3 (mean**)	-	-	-	-	**4.2**	**2.0**	**0.596**	**0.444**	**0.377**	**-0.185**	**-0.115**	**0.495**
*Gg15*	(GA)_n_	0.2	FAM	147-169	3	2.05	0.977	0.514	0.392	-0.910**	-0.308	0.370
*Gg17*	(AC)_n_	0.2	PET	159-181	3	1.02	0.023	0.023	0.023	-0.003**	0.000	1.000
*Gg18*	(GA)_n_	0.2	VIC	179-187	6	2.24	0.976	0.558	0.456	-0.759**	-0.272	0.776
*Gg22*	(GT)_n_	0.2	FAM	237-247	4	2.25	0.964	0.559	0.455	-0.733**	-0.263	0.488
*Gg23*	(AC)_n_	0.2	VIC	258-278	6	2.84	1.000	0.651	0.582	-0.540**	-0.214	0.562
**MPS4 (mean)**	-	-	-	-	**4.4**	**2.08**	**0.788**	**0.461**	**0.3816**	**-0.589**	**-0.211**	**0.615**
**Overall (mean)**	**-**	**-**	**-**	**-**	**5.7**	**2.4**	**0.691**	**0.512**	**0.444**	**0.010**	**-0.139**	**0.561**

Primer concentration in the final reaction as μM/primer ([P]); Number of alleles per locus (*A*_*N*_); effective number of alleles (*A*_*E*_); observed heterozygosity (*H*_*O*_); expected heterozygosity (*H*_*E*_); polymorphic information content (*PIC*); inbreeding coefficient (*F*_*IS*_) with statistically significant deviations from Hardy-Weinberg expectations indicated by *(*P* < 0.01) and **(*P* < 0.001); null allele frequency (*Fr*_*NULL*_) with ^b^indicating the presence of null alleles at statistical significance at the 5% nominal level and Ewans-Watterson probability (*P*_*E-W*_). Mean values for each multiplex assay and overall are indicated in bold.

### Multiplex assay cross-species amplification and efficiency in species identification

Cross-species amplification of the four multiplex assays was tested for 11 other southern African shark species (Table 3). The number of alleles observed was highest in *G. galeus* and *M. palumbes,* varying from 1 to 7, while the percentage of polymorphism (*PP*) for each marker ranged from 31.8%-95.5%. The polymorphic information content (*PIC*) decreased exponentially with evolutionary distance from the source species (Table 3) and the four multiplex assays showed the highest *PIC* in *M. mustelus*, *M. palumbes* and *G. galeus*.

**Table 3:** Multiplex transferability results of a total of 22 microsatellite loci showing the number of alleles per locus for the 11 elasmobranch species tested

**Locus**	**MP**	**GG**	**CB**	**CL**	**CO**	**CP**	**HP**	**HE**	**SL**	**SZ**	**PP**
	***(n = 8)***	***(n = 8)***	***(n = 8)***	***(n = 4)***	***(n = 4)***	***(n = 4)***	***(n =8)***	***(n = 4)***	***(n = 5)***	***(n = 5)***	***(n = 5)***
**MPS1**											
*Mh1*	4	3	1	2	1	1	1	1	4	2	3
*Mh2*	-	5	*	1	1	*	-	-	2	1	3
*Mh9*	4	4	*	-	*	*	-	-	**4**	5	**3**
*Mh25*	5	5	2	3	2	3	1	3	**4**	6	3
*Mca25*	3	1	1	3	2	1	2	1	**3**	**4**	3
*McaB39*	**3**	3	2	2	1	2	2	*	2	**3**	3
**MPS2**											
*McaB5*	3	1	2	4	2	3	1	1	**5**	**5**	7
*McaB6*	4	4	*	4	2	1	-	1	2	**3**	**5**
*McaB22*	2	1	1	4	2	4	2	4	**4**	7	7
*McaB27*	2	2	2	-	**1**	*	-	1	-	2	-
*Mca33*	4	2	2	4	2	2	2	3	**4**	**7**	6
*McaB37*	3	5	**1**	3	**1**	**1**	-	-	**4**	**6**	**7**
**MPS3**											
*Gg2*	5	4	1	-	**1**	**1**	2	-	-	2	-
*Gg3*	3	2	1	1	2	*	2	1	-	2	**2**
*Gg7*	4	1	1	-	1	1	**2**	-	-	1	1
*Gg11*	6	4	1	-	2	2	*	*	-	1	3
*Gg12*	4	5	-	-	1	*	1	**1**	2	2	**1**
**MPS4**											
*Gg15*	7	5	1	4	1	4	4	2	5	3	5
*Gg17*	4	4	1	4	1	2	**2**	2	2	3	2
*Gg18*	6	3	2	3	1	3	3	**3**	7	6	4
*Gg22*	6	5	2	3	2	2	2	*1*	3	2	3
*Gg23*	4	2	2	1	2	1	**3**	**2**	6	4	3
**Total P loci**	21	18	8	13	10	10	12	7	16	19	18
***PP***	95.5	81.8	36.4	59.1	45.5	45.5	54.5	31.8	72.7	86.3	81.8

*n* - number of individuals tested; *Failed to amplify but showed successful transferability initially (see Table 1); - No amplification; allele numbers in bold indicate loci that failed to cross-amplify according to Table 1; P – polymorphic and *PP* - percentage of polymorphism. For species abbreviations refer to Table 1.

**Figure 4:**
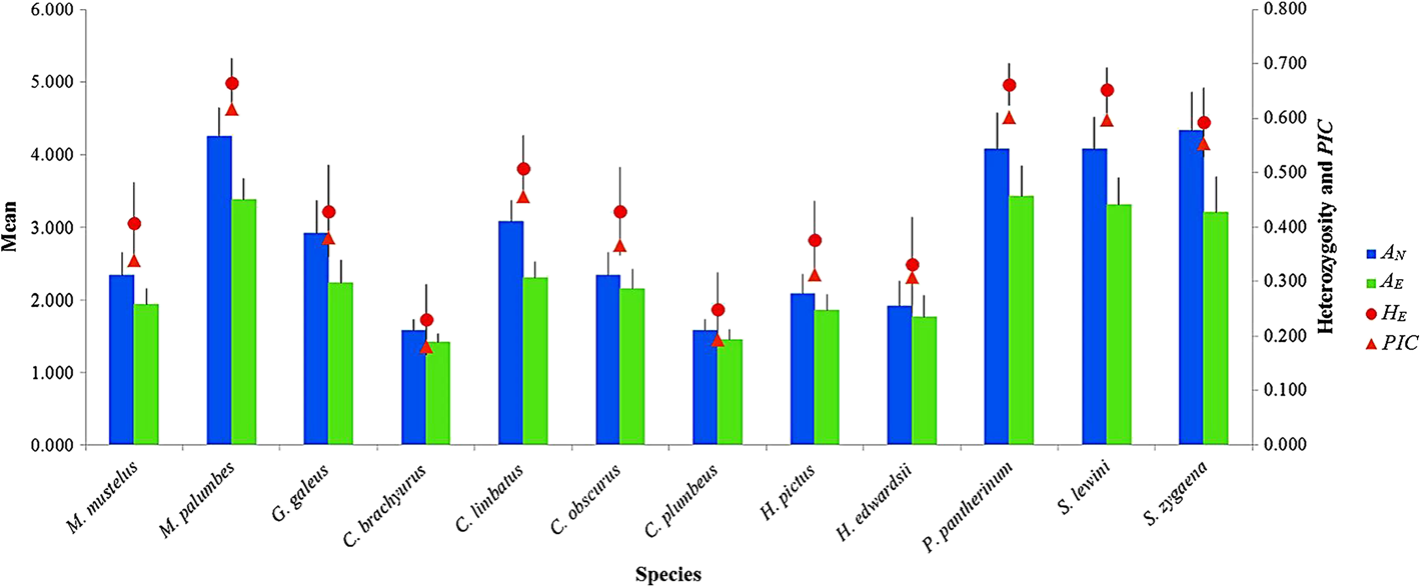
**Mean genetic diversity estimates using 12 microsatellite loci shared between species: number of alleles (*A***_***N***_**), effective number of alleles (*A***_***E***_**), heterozygosity (*H***_***E***_**) and polymorphic information content ( *PIC *). Error bars represent standard error.**

The mean genetic diversity estimates for each species in terms of number of alleles (*A*_*N*_), effective number of alleles (*A*_*E*_), observed heterozygosity (*H*_*O*_), expected heterozygosity (*H*_*E*_) and *PIC* are shown in Figure 4. In group 1, the mean *H*_E_, *A*_E_ and *PIC* varied from relatively low in *C. brachyurus* (mean *H*_E_ = 0.230; mean *A*_E_ = 1.4; mean *PIC* = 0.181) to relatively high in *M. palumbes* (mean *H*_E_ = 0.653; mean *A*_E_ = 3.3; mean *PIC* = 0.606). Group 2 exhibited similar patterns of genetic diversity that varied from moderate in *S. zygaena* (mean *H*_E_ = 0.593; mean *A*_E_ = 3.2; mean *PIC* = 0.554) to relatively high in *P. pantherinum* (mean *H*_E_ = 0.662; mean *A*_E_ = 3.4; mean *PIC* = 0.603). For group 3 with *n* = 4, the mean *H*_E_, *A*_E_ and *PIC* ranged from relatively low in *C. plumbeus* (mean *H*_E_ = 0.249; mean *A*_E_ = 1.5; mean *PIC* = 0.193) to relatively high in *C. obscurus* (mean *H*_E_ = 0.429; mean *A*_E_ = 2.1; mean *PIC* = 0.367).

**Figure 5:**
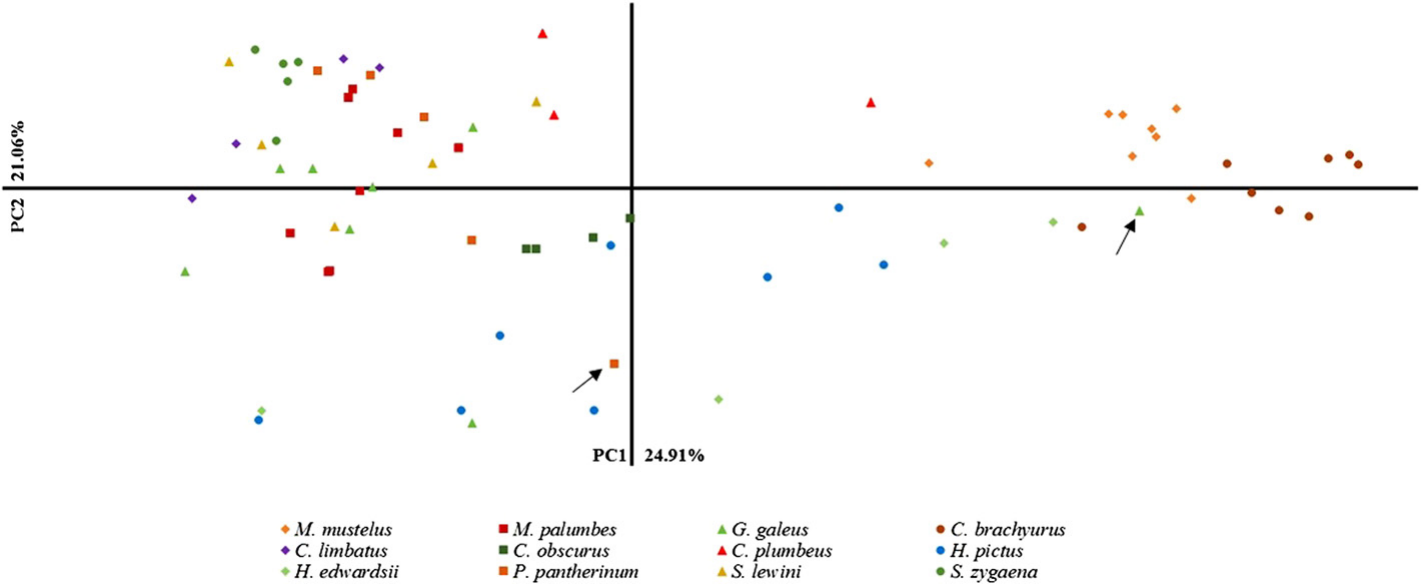
**Principle coordinates analysis (PCoA) of study species based on 12 shared amplified microsatellite loci between species.** Arrows depict misidentified/mislabelled individuals.

The genotypic distribution of the study species is depicted in Figure 5. Most of the study species could be differentiated on PC1 and PC2 of the PCoA plot as can be seen from individuals of each respective species clustering together. Individuals of the catshark species (*H. edwardsii*, *P. pantherinum* and *H. pictus*) however, were dispersed across quadrant 3 and 4. The PCoA also revealed that one of the *M. mustelus* individuals was misidentified as *G. galeus*. The identity of this particular individual was subsequently confirmed using the genetic identification method developed specifically for smoothhound sharks [16]. Briefly, this method involves using four primers (1 universal forward primer and 3 species-specific reverse primers) for the mitochondrial gene, *nicotinamide adenine dehydrogenase subunit 2* (*ND2*), in a multiplex PCR reaction. The reverse primers amplify a fragment of different length for each species (*M. asterias*, 564 bp; *M. mustelus*, 392 bp; *G. galeus*, 671 bp) and can therefore be utilised for distinguishing species based on fragment size.

In this study, the potential use of microsatellite loci in species identification was successfully demonstrated using shared microsatellite loci between species. The polymorphic information of these microsatellite loci was characterised by low genetic variation as previously proposed for elasmobranchs [31]. The genotypic distribution of the study species could also be differentiated based on PCoA analysis. Markedly, the lack of differentiation between the catshark species (*H. edwardsii* and *H. pictus*) on the PCoA plot may be explained by the misidentification of the *Haploblepharus* species that is a common occurrence in the catshark family [54]. To further investigate whether the lack of differentiation detected with microsatellites was indeed due to misidentification, the *cytochrome b* (*Cyt b*) and *ND2* genes were applied for species comparisons [54]. For both genes, sequence analyses revealed individuals with cryptic identification, suggesting that in the case of the catsharks, the microsatellites optimised in the current study were not successful in discriminating between the *Haploblepharus* species but could in the future aid in the identification of cryptic speciation within the catshark family.

In South Africa, the aforementioned misidentification issue is prominent in fishing operations (particularly in longline and trawl fisheries) where there is a high rate of incidental capture of non-target shark species [9-12]. This hinders the collection of reliable data on shark catch and trade on a species-specific basis making robust stock assessments and identification of overfished and potentially threatened species nearly impossible in most situations [4]. This was apparent in a study by Attwood *et al.*[12], which assessed bycatch in South Africa’s inshore trawl fishery based on observer records. In the aforementioned study, certain taxa were difficult to identify, and therefore taxonomic groups were lumped in species groups (*e.g. Raja* spp., *Mustelus* spp. and Scyliorhinidae), even though every attempt was made to analyse data at species level. Therefore, the molecular genetic makers developed by the current study may facilitate in obtaining species-specific catch data for stock assessment, characterising genetic diversity and delineating population genetic structure. This in turn will contribute to the implementation of future conservation and management plans on a species-specific level in southern Africa.

## Conclusions

Cross-species amplification of available microsatellite loci to target species has proven to be more time- and cost-effective in comparison to the *de novo* development approach and permitted the cross-amplification of 22 markers across 12 elasmobranch species. Cross-species amplification of the four multiplex assays developed in the current study highlighted the usefulness of microsatellites for characterising genetic diversity and potentially also species identification of a number of commercially important and endemic elasmobranch species. The molecular genetic markers developed in this study and their usefulness in down-stream applications could therefore aid in a more integrative, multidisciplinary approach to conservation management of elasmobranchs in southern Africa.

## Methods

### Ethics statement

The collection of specimens from various shark species used in this study complied with the Convention on Biological Diversity (http://www.cbd.int/convention/) and the Convention on the Trade in Endangered Species of Wild Fauna and Flora (http://www.cites.org/). All permits to collect finclip or muscle tissue for research purposes were granted by the Department of Agriculture, Forestry and Fisheries (Republic of South Africa).

### Study species and DNA extraction

Sixteen elasmobranch species occurring in southern African waters belonging to five families within two orders were selected for cross-species amplification (Additional file 1). Where possible, specimens were collected from at least two sampling locations to better capture allelic diversity present within populations of each respective species. However, due to opportunistic sampling for a majority of the study species, samples were obtained from only a single location (Additional file 1). Muscle tissue or finclips were preserved in 99% ethanol and stored at room temperature until further use. Total genomic DNA was isolated using the standard cetyltrimethylammonium bromide (CTAB) method of Saghai-Maroof *et al*. [55]. The extracted DNA was quantified using a NanoDrop ND-1000 spectrophotometer v.3.0.1 (*NanoDrop®*). For testing cross-species amplification, each DNA sample was adjusted to a working concentration of 50 ng/μl and stored at -20°C prior to polymerase chain reaction (PCR) analysis.

### Microsatellite primer transfer

A total of 35 microsatellite markers previously developed in five elasmobranch species (*Raja pulchra*[44], *Scyliorhinus canacula*[45], *M. canis*[49], *G. galeus*[50] and *M. henlei*[56,57]) were selected for testing cross-species amplification. Primer sequences and annealing temperature (T_A_) of each primer set optimised for each respective source species are shown in Table S2 (Additional file 2). Polymerase chain reaction conditions optimised for the majority of the source species [*G. galeus*, *M. henlei*, *R. pulchra* and *S. canacula*] were applied for cross-species microsatellite examinations except for the *M. canis* ( [56] PCR protocol) and some *R. pulchra* (modified [57] PCR protocol) primer sets. Polymerase chain reactions for all individuals were executed in a GeneAmp® PCR System 2700.

The PCR amplicons were visualised on a 2% agarose gel stained with ethidium bromide together with negative controls and Promega 100 bp molecular size ladder for preliminary size determination. Success or failure of PCR amplification in cross-species trials was determined simply on the basis of whether band intensity was sufficient to score alleles. In most instances, less stringent PCR conditions were not employed in the cross-species assays so as to minimise the risk of amplification of non-orthologous loci in the target species. The number of markers that showed amplification success at all or a percentage of individuals in the target species (“+/++” in Table 1) were counted as an index to measure the cross-species microsatellite amplification performance.

### Multiplex design and optimisation

As this study forms part of a larger project on the development of genetic resources for commercially important and endemic species of southern Africa, *Mustelus mustelus* was used as a candidate species for testing of the four novel multiplex assays. Levels of polymorphism were initially assessed at all the successfully cross-amplified microsatellite loci in a panel of eight individuals of *M. mustelus*. Amplicons were subjected to electrophoresis for two hours at 150 volts on a 12% polyacrylamide gel to detect size variants. Microsatellites were considered to be polymorphic when two bands were distinguishable in a single individual (*i.e.* heterozygote) and/or clear size differences were detected between different individuals.

Twenty-two polymorphic microsatellite loci were selected, and primers fluorescently labelled and optimised in four multiplex assays (5–6 loci per MPS) using a strategy outlined by Guichoux *et al*. [58] with one of the following dyes: FAM, VIC, PET, or NED. The use of different dyes was to facilitate co-amplification of multiple microsatellite markers in a single reaction for cost- and time-efficient genotyping (Multiplex PCR).

After optimisation of the newly designed MPS (MPS1, MPS2, MPS3 and MPS4), a panel of 87 *M. mustelus* individuals from across the distribution range in southern Africa was genotyped for marker characterisation purposes. The multiplex assays were then tested on 11 additional species to show their overall application in genetic diversity and population structure analysis. Finally, a total of 12 microsatellite loci that were successfully genotyped across the study species (*Mh1*, *Mh25*, *Mca25*, *McaB39*, *McaB5*, *McaB22*, *Mca33*, *Gg15*, *Gg17*, *Gg18*, *Gg22* and *Gg23*) were selected to demonstrate the potential use of microsatellite loci in species identification.

The percentage of polymorphism (*PP*) was calculated using the formula:

$$\mathrm{PP}=\frac{N_{P}}{N_{T}}\;X\;100$$

where *N*_*P*_ is the total number of polymorphic loci and *N*_*T*_ is the total number of loci multiplied by 100.

For the multiplex reaction, the Qiagen Multiplex PCR kit was used and PCR conducted according to the manufacturer’s instructions except for varying T_A_, 59°C for MPS1, MPS3 and MPS4; and 56°C for MPS2. For subsequent analysis on an ABI 3730XL DNA Analyzer, PCR products were diluted in distilled water and fragment analysis performed together with the LIZ600 internal size standard. Individual genotypes were scored based on fragment size via Peak Scanner® software v.1 (Life Technologies). AutoBin v.0.9 macro for Excel (http://www.bordeaux-aquitaine.inra.fr/biogeco/Ressources/Logiciels/Autobin; see [58]) was used to detect discreet size variants where allele binning of genotype data obtained from Peak Scanner® software v.1 was based on raw size.

### Genetic diversity analysis

MicroChecker v.2.2.3 [59] was used to evaluate the presence of genotypic errors caused by allele dropout, stuttering and null alleles. Null allele frequencies (*Fr*_*NULL*_) were calculated using the Brookfield 1 estimator implemented in this program. Locus-specific fixation index (*F*_*IS*_) and over all loci was estimated to measure departure from Hardy-Weinberg equilibrium using the exact probability test (20 batches, Dememorization; 10000 and 5000 iterations) using Genepop v.4.0 [60]. Linkage disequilibrium between all pairs of loci was calculated using an exact test implemented also in Genepop. Slatkin’s exact test (1000 permutations) for neutrality, based on Ewens-Watterson sampling theory [61] was used to detect loci under selection as implemented in Arlequin v.3.5.1.2 [62]. The number (*A*_*N*_) of alleles at each microsatellite locus, as well as the effective number of alleles *A*_*E*_:

$$A_{E}=1/\sum_{i=1}^{n}{p_{i}}^{2}$$

where *p*_*i*_ is the frequency of the *i*^th^ allele and *n* is the number of alleles was calculated using the GenAlEx v.6.5 program [63]. The proportion of individual samples that were heterozygous [direct count heterozygosity (*H*_*O*_) and expected under Hardy-Weinberg equilibrium (*H*_*E*_)] was calculated using MsatTools [64]. MsatTools was also used to calculate the polymorphic information content (*PIC*) of each marker according to the following equation in [65]:

$\;\mathrm{PIC}=1-\sum_{i=1}^{n}{p_{i}}^{2}-2\left[\sum_{i=1}^{n-1}\sum_{j=i+1}^{n}{p_{i}}^{2}{p_{j}}^{2}\right]$ where *p*_*i*_ and *p*_*j*_ are the frequency of the *i*^th^ and *j*^th^ allele respectively and *n* is the number of alleles.

Direct comparison of genetic diversity estimates (*H*_E,_*A*_*E*_ and *PIC*) across the 11 species was not plausible due to the different sample sizes that were used. Species were therefore grouped into three groups according to sample size: (1) *M. mustelus*, *M. palumbes, G. galeus*, *C. brachyurus* and *H. pictus* (*n* = 8); (2) *P. pantherinum*, *S. lewini* and *S. zygaena* (*n* = 5) and (3) *C. obscurus*, *C. limbatus, C. plumbeus* and *H. edwardsii* (*n* = 4). The potential use of microsatellite data for species-assignment was assessed through principle coordinate analysis (PCoA) in GenAlEx v.6.5 using genetic distances between individuals.

To evaluate cross-species amplification performance, DNA sequences derived from the mitochondrial *ND2* gene (1044 bp**)** of each species were downloaded from GenBank and Global Cestode Database: Elasmobranchs Specimens (http://elasmobranchs.tapewormdb.uconn.edu) (Additional file 3). The genetic distance of the study taxa was estimated using the Kimura 2-parameter model with the rate variation among sites modelled with a gamma distribution (shape parameter = 5) implemented in MEGA v.5 [66].

## Abbreviations

CI: Confidence interval; *PP*: Percentage of polymorphism; *P*: Polymorphic; *PIC*: Polymorphic information content; *A*_*N*_: Number of alleles; *A*_*E*_: Effective number of alleles; *H*_*O*_: Observed heterozygosity; *H*_*E*_: Expected heterozygosity; *F*_*IS*_: Inbreeding coefficient; *Fr*_*NULL*_: Null allele frequency; *P*_*E-W*_: Ewans-Watterson probability; K2P: Kimura 2-parameter model; *ND2*: *Nicotinamide adenine dehydrogenase subunit 2*; MM: *Mustelus mustelus*; MP: *Mustelus palumbes*; GG: *Galeorhinus galeus*; SQ: *Scylliogaleus quecketti*; CB: *Carcharhinus brachyurus*; CL: *Carcharhinus limbatus*; CO: *Carcharhinus obscurus*; CP: *Carcharhinus plumbeus*; HP: *Haploblepharus pictus*; HE: *Haploblepharus edwardsii*; PA: *Poroderma africanum*; PP: *Poroderma pantherinum*; SL: *Sphyrna lewini*; SZ: *Sphyrna zygaena*; RS: *Raja straeleni*; RA: *Raja alba*.

## Competing interests

The authors have no competing interests to declare.

## Authors’ contributions

SNM performed cross-species amplification, microsatellite genotyping, genetic data analyses, organised the samples and drafted the manuscript. CR participated in cross-species amplification and microsatellite genotyping. RR-W participated in experimental design and coordination and contributed to manuscript preparation. AEB-vdM conceived the study, provided funds, participated in its design and coordination and contributed to manuscript preparation. All authors read and approved the final manuscript.

## Supplementary Material

Additional file 1 The 16 elasmobranch species of southern Africa selected for cross-species amplification, including family, species, distribution and sampling locations. Click here for file

Additional file 2 **The 35 putative microsatellite markers developed from five closely related species for cross-species amplification in the study taxa, including the primers sequence, microsatellite repeat motif, annealing temperature (T**_**A**_**) and GenBank accession numbers.** Click here for file

Additional file 3 **The *ND2 *** **sequence information of the study taxa used to estimate the genetic distance to evaluate cross-species performance, including ID Verified, availability of images (yes or no) which are available in the on-line host specimen database (**http://elasmobranchs.tapewormdb.uconn.edu) and GenBank accession numbers. Click here for file
