# Supplementary material for: Microsatellite cross-species amplification and utility in southern African elasmobranchs: A valuable resource for fisheries management and conservation
Source: BMC Res Notes. 2014 Jun 10;7:352. doi: 10.1186/1756-0500-7-352 (PMC4079218; doi:10.1186/1756-0500-7-352)
Supplement: Additional file 1 — The 16 elasmobranch species of southern Africa selected for cross-species amplification, including family, species, distribution and sampling locations. [file 1756-0500-7-352-S1.doc]

**Additional file 1:** The table presents 16 elasmobranch species of southern Africa selected for cross-species amplification, including family, species/vernacular, distribution and sampling locations.

Table S1. The 16 elasmobranch species of southern Africa selected for cross-species amplification, including family, species, distribution and sampling locations.

*^a^* [67]

| **Family** | **Species: common name** | **Distribution*^a^*** | **N** | **Sample Location** |
| --- | --- | --- | --- | --- |
| ***Order Carcharhiniformes*** |  |  |  |  |
| Triakidae | *Mustelus mustelus*: Common smoothhound/ houndshark | 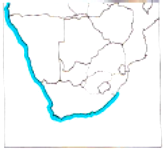 | 8 | Angola (2), False Bay (4) and Durban (4) |
|  | *Mustelus palumbes*: Whitespotted smoothhound shark | 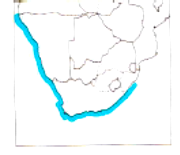 | 8 | Mossel Bay, Struisbaai |
|  | *Galeorhinus galeus*: Tope shark | 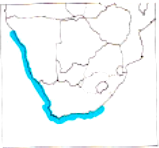 | 8 | Robben Island (2), Langebaan Lagoon (4) and False Bay (2) |
|  | *Scylliogaleus quecketti:* Flapnose houndshark | 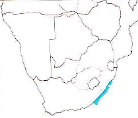 | 1 | Mossel Bay |
| Carcharhinidae | *Carcharhinus brachyurus*: Bronze whaler/ copper shark | 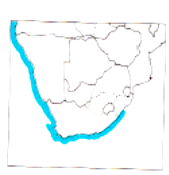 | 5 | Durban |
|  | *Carcharhinus limbatus*: Blacktip shark | 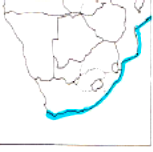 | 4 | Mossel Bay |
|  | *Carcharhinus obscurus*: Dusky shark | 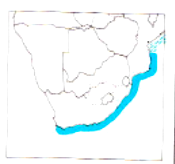 | 8 | Durban |
|  | *Carcharhinus plumbeus*:  Sandbar shark | 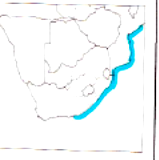 | 5 | Durban |
| Scyliorhinidae | *Haploblepharus edwardsii*: Puffadder/ Happy eddie shyshark | 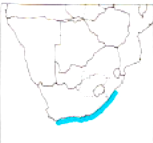 | 4 | Hermanus |
|  | *Haploblepharus pictus*: Dark shyshark | 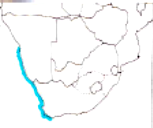 | 8 | Hermanus |
|  | *Poroderma africanum*: Pyjama shark | 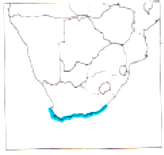 | 1 | Hermanus |
|  | *Poroderma  pantherinum*: Leopard catshark | 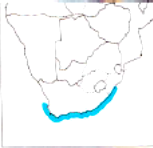 | 5 | Hermanus (2), Mossel Bay (3) |
| Sphyrnidae | *Sphyrna lewini*: Scalloped hammerhead | 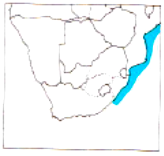 | 5 | Mossel Bay |
|  | *Sphyrna zygaena*: Smooth hammerhead | 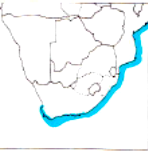 | 5 | Mossel Bay |
| ***Order Rajiformes*** |  |  |  |  |
| Rajidae | *Raja (Rostroraja) alba*: Spearnose skate | 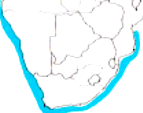 | 3 | Port Elizabeth (1), Mossel Bay (2) |
|  | *Raja straeleni*: Biscuit skate | 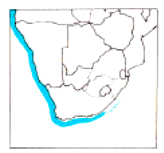 | 1 | West Coast |
