# Supplementary material for: Microsatellite cross-species amplification and utility in southern African elasmobranchs: A valuable resource for fisheries management and conservation
Source: BMC Res Notes. 2014 Jun 10;7:352. doi: 10.1186/1756-0500-7-352 (PMC4079218; doi:10.1186/1756-0500-7-352)
Supplement: Additional file 2 — The 35 putative microsatellite markers developed from five closely related species for cross-species amplification in the study taxa, including the primers sequence, microsatellite repeat motif, annealing temperature (T A ) and GenBank accession numbers. [file 1756-0500-7-352-S2.doc]

**Additional file 2:** The table presents 35 putative microsatellite markers developed from five closely related species for cross-species amplification in the study taxa, including the primers sequence, microsatellite repeat motif, annealing temperature (T_A_) and GenBank accession numbers.

Table S2. The 35 putative microsatellite markers developed from five closely related species for cross-species amplification in the study taxa, including the primers sequence, microsatellite repeat motif, annealing temperature (T_A_) and GenBank accession numbers.

| **Locus** | **Primer sequence (5’-3’)** | **Microsatellite repeat motif** | **T_A_** | **Accession number** | **References** |
| --- | --- | --- | --- | --- | --- |
| *Mh1* | F: GGAGGAGGGAAGCCTATGG  R: TCTCTGGCTCCATTCAGGG | (AG)_n_ | 59 | N/A | [56] |
| *Mh2* | F: ACTACACTGCATATAAACAGGC  R: TTTTCAGAGGGCATAACTCAC | (GA)_n_ | 56 | N/A | [57] |
| *Mh6* | F: CATGTCCACTTCCCATCGC  R: GGAGAGATTAGAACAGGTGGC | (CT)_n_ | 59 | N/A | [56] |
| *Mh9* | F: CAACCATCTTTACTACACTG  R: GATGGACCTCACATTTAACAC | (GA)n | 56 | N/A | [57] |
| *Mh25* | F: TGCAATAACCGTTCTGCGTC  R: TCACACCCGCAGTTAGATCC | (CT)_n_ | 59 | N/A | [56] |
| *Mca25* | F: ACACACTTTCACGCACAAGC  R: TCGCTCAAGTGAGACCAGAG | (CA)_n_(CT)_n_ | 59 | JN129145 | [49] |
| *Mca31* | F: GGCAGATCAGTTGAGGAAGG  R: AATGGGGAGACTTCTCTTTGC | (ATC)_n_ | 59 | JN083992 | [49] |
| *Mca33* | F: CATTTGAACCCCGACAGAAC  R: TCCAAGTAAGGATGAGTGACACC | (ATC)_n_ | 59 | JN083993 | [49] |
| *Mca44* | F: TTTCCGCTGTATCACACATACAC  R: GCATCTATATGTCTGCGTGTGTC | (AC)_n_ | 59 | JN083995 | [49] |
| *McaB5* | F: TAATCGACACGCAGTCATCG  R: AAGCTCCAATTCTCACTGTGC | (GT)_n_ | 59 | JN083996 | [49] |
| *McaB6* | F: AGGATAAATACACGCACACAGG  R: TTTTTGTTTTGCAATCTCACG | (CA)_n_ | 59 | JN083997 | [49] |
| *McaB22* | F: TCCTCTCCAGGACAAACACAC  R: TCCCACCTGCCATAGTAATTG | (AC)_n_ | 59 | JN083999 | [49] |
| *McaB27* | F: ATCCAGTGGTTTTGAAATGC  R: CCTCGTAGGTCTCGTC | (GT)_n_ | 59 | JN129154 | [49] |
| *McaB33* | F: TCTCCTAATGGAACGTGTGC  R: GGTATGCGTATGGGTGTCG | (CA)_n_ | 59 | JN084002 | [49] |
| *McaB35* | F: AGTGCGTGCCAGTGTATGAG  R: GTTCTGCATGGGACGTGAC | (TG)_n_ | 59 | JN084003 | [49] |
| *McaB37* | F: TCTGCCTCTGTGTCTCATCC  R: TTTCCATTTCCGACATAGGG | (GT)_n_ | 59 | JN084005 | [49] |
| *McaB39* | F: GGACAGGCAGCATCTGTGTA  R: CCCAGGGGGATTAGGATATT | (CA)_n_GAT(AC)_n_ | 59 | JN129156 | [49] |
| *Gg2* | F: TGGCTCAGTCCAGAAACCC  R: CCCTATTCGAGAGGCCCAG | (TG)_n_ | 59 | N/A | [50] |
| *Gg3* | F: CCGTGACTGAAAGCAGCC  R: CCCTCAACCATGGCAAGTG | (GATT)_n_ | 59 | N/A | [50] |
| *Gg7* | F: CTGTGGAACCAAACTCCAGC  R: AGCTGGTCGAGGTGAATGC | (AG)_n_ | 59 | N/A | [50] |
| *Gg11* | F: AAGTTGCACGTTTCCCAGC  R: TACTGCAGGACCGGTTTCC | (TCCC)_n_ | 59 | N/A | [50] |
| *Gg12* | F: TGTCAAACACCATCGCAGG  R: TGCTCTGAAGTCTACAAGAATGG | (TA)_n_ | 59 | N/A | [50] |
| *Gg15* | F: GGCTGAATGGTTTCCCAGC  R: GCCTCCAACTTAGCATAGCC | (GA)_n_ | 59 | N/A | [50] |
| *Gg17* | F: CCTGCTTGTGACAGTTACCC  R: ACAGGCATCACCTCTGTGC | (AC)_n_ | 59 | N/A | [50] |
| *Gg18* | F: TCCACTTCAGGAAGGCCAG  R: CAAAGCCAGGTGGTTCTCC | (GA)_n_ | 59 | N/A | [50] |
| *Gg22* | F: TCCTGGGATGGCAACTTCG  R: AGGCCACCCAACTATCCTG | (GT)_n_ | 59 | N/A | [50] |
| *Gg23* | F: ACAGACCACAGGGCATGG  R: TGCAGAGCAGGCTAGATGG | (AC)_n_ | 59 | N/A | [50] |
| *Rp16-nfrdi* | F: AGGAAGGCTTCAGCACATAAT  R: CTCATCTGGAAGAGCACACAC | (TG)_13_ | 54 | JQ433557 | [44] |
| *Rp35-nfrdi* | F: CTTACTGGTGAGGAATCTGAGC  R: GCATACACTCCACACACCAC | (TG)_9_ | 61 | JQ433564 | [44] |
| *Scan02* | F: TGCAGCTTCGCTATCTTATGC  R: AAATCTGCTGCTCGCTTCAT | (TG)_9_ | 60 | N/A | [45] |
| *Scan06* | F: GGCAGTGATTGCATTCTTGA  R: CAGAAACTGTGCAGAAATCACA | (TG)_9_ | 60 | N/A | [45] |
| *Scan12* | F: GCCAGTGGCTATAACGGA AC  R: TCC CAC ACA GTC CTG TTGAA | (AG)_9_ | 60 | N/A | [45] |
| *Scan14* | F: AACCATCCTCCGCAAATAAA  R: GAACAGTGCCCCAAGTTCAT | (CA)_9_ | 60 | N/A | [45] |
| *Scan15* | F: TCATCATCATCACCACCAGAA  R: GAGCTATGCTGGCAATTCGT | (CA)_15_ | 60 | N/A | [45] |
| *Scan16* | F: CCGACTCCTTTGGATGTGTT  R: GGACGCTCTCGTTCTTATGC | (TG)_9_ | 60 | N/A | [45] |
