# Supplementary material for: Microsatellite cross-species amplification and utility in southern African elasmobranchs: A valuable resource for fisheries management and conservation
Source: BMC Res Notes. 2014 Jun 10;7:352. doi: 10.1186/1756-0500-7-352 (PMC4079218; doi:10.1186/1756-0500-7-352)
Supplement: Additional file 3 — The ND2 sequence information of the study taxa used to estimate the genetic distance to evaluate cross-species performance, including ID Verified, availability of images (yes or no) which are available in the on-line host specimen database (http://elasmobranchs.tapewormdb.uconn.edu) and GenBank accession numbers. [file 1756-0500-7-352-S3.doc]

**Additional file 3:** The table presents the *ND2* sequence information of the study taxa used to estimate the genetic distance to evaluate cross-species performance, including ID Verified, availability of images (yes or no) which are available in the on-line host specimen database ([http://elasmobranchs.tapewormdb.uconn.edu](http://elasmobranchs.tapewormdb.uconn.edu/)) and GenBank accession numbers.

complied with the Convention on Biological Diversity ([http://www.cbd.int/convention/](https://webmail.sun.ac.za/owa/redir.aspx?C=bhhppx3I4kCPa2s0OaZrDarU3gLiTdEImZH9_OL7LT62zWfn1GsXHe6d9yLQ5PUBTv1XsyeRoBw.&URL=http%3A%2F%2Fwww.cbd.int%2Fconvention%2F)) and the Convention on the Trade in Endangered Species of Wild Fauna and Flora([http://www.cites.org/](https://webmail.sun.ac.za/owa/redir.aspx?C=bhhppx3I4kCPa2s0OaZrDarU3gLiTdEImZH9_OL7LT62zWfn1GsXHe6d9yLQ5PUBTv1XsyeRoBw.&URL=http%3A%2F%2Fwww.cites.org%2F))

Table S3: The *ND2* sequence information of the study taxa used to estimate the genetic distance to evaluate cross-species performance, including ID Verified, availability of images (yes or no) which are available in the on-line host specimen database ([http://elasmobranchs.tapewormdb.uconn.edu](http://elasmobranchs.tapewormdb.uconn.edu/)) and GenBank accession numbers.

| **Genus** | **Species** | **ID Verified** | **No. Images** | **GN No.** | **GenBank No.** | **Unique ID** |
| --- | --- | --- | --- | --- | --- | --- |
| *Mustelus* | *mustelus* | Yes | 5 | 7218 | JQ518709.1 | 5963 |
| *Mustelus* | *palumbes* | Yes | 5 | 7322 | JQ518710.1 | 6067 |
| *Mustelus* | *canis* | ? | ? | 917 | JQ518711.1 | ? |
| *Galeorhinus* | *galeus* | Yes | 5 | 7236 | JQ518695.1 | 5981 |
| *Scylliogaleus* | *quecketti* | ? | ? | - | DQ422121.1 | - |
| *Carcharhinus* | *plumbeus* | ? | ? | 903 | JQ518632.1 | ? |
| *Carcharhinus* | *brachyurus* | ? | ? | 3 | JQ518611.1 | ? |
| *Carcharhinus* | *obscurus* | Yes | 5 | 3213 | JQ518612.1 | 4679 |
| *Carcharhinus* | *limbatus* | ? | ? | 1303 | JN082204.1 | ? |
| *Haploblepharus* | *edwardsii* | Yes | 5 | 7237 | JQ518679.1 | 5982 |
| *Poroderma* | *africanum* | ? | ? | 1772 | JQ518682.1 | ? |
| *Poroderma* | *pantherinum* | Yes | 5 | 7325 | JQ518683.1 | 6070 |
| *Sphyrna* | *lewini* | ? | ? | 5663 | JQ518691.1 | ? |
| *Sphyrna* | *zygaena* | ? | ? | 1097 | JQ519079.1 | ? |
| *Raja* | *straeleni* | Yes | 5 | 7192 | JQ518894.1 | 5937 |
| *Rostroraja* | *alba* | Yes | 5 | 7302 | JQ518900.1 | 6047 |
